# Low-density lipoprotein cholesterol and risk of hepatocellular carcinoma: a Mendelian randomization and mediation analysis

**DOI:** 10.1186/s12944-023-01877-1

**Published:** 2023-07-31

**Authors:** Jiali Cao, Ziwen Wang, Mengpei Zhu, Yumei Huang, Ze Jin, Zhifan Xiong

**Affiliations:** Department of Gastroenterology, Liyuan Hospital, Tongji Medical College of Huazhong University of Science and Technology, Wuhan, 430077 China

**Keywords:** Low-density lipoprotein cholesterol, Hepatocellular carcinoma, Coronary artery disease, Mendelian randomization

## Abstract

**Background:**

A previous study demonstrated that low-density lipoprotein cholesterol (LDL-C) is associated with hepatocellular carcinoma (HCC); however, the causality between them has not been proven due to conflicting research results and the interference of confounders. This study utilized Mendelian randomization (MR) to investigate the causal relationship between LDL-C and HCC and identify the mediating factors.

**Methods:**

LDL-C, HCC, and coronary artery disease (CAD) genome-wide association study (GWAS) data were obtained from a public database. To investigate causality, inverse variance weighting (IVW) was the main analysis approach. MR‒Egger, simple mode, weighted median (WM), and weighted mode were employed as supplementary analytic methods. In addition, horizontal pleiotropy and heterogeneity were tested. To evaluate the stability of the MR results, a "leave-one-out" approach was used. Multivariate MR (MVMR) was utilized to correct the confounders that might affect causality, and mediation analysis was used to investigate the potential mediating effects. Finally, we used HCC risk to infer the reverse causality with LDL-C level.

**Results:**

Random effects IVW results were (LDL-C-HCC: odds ratio (OR) = 0.703, 95% confidence interval (CI) = [0.508, 0.973], *P* = 0.034; CAD–HCC: OR = 0.722, 95% CI = [0.645, 0.808], *P* = 1.50 × 10^–8^; LDL-C–CAD: OR = 2.103, 95% CI = [1.862, 2.376], *P* = 5.65 × 10^–33^), demonstrating a causal link between LDL-C levels and a lower risk of HCC. Through MVMR, after mutual correction, the causal effect of LDL-C and CAD on HCC remained significant (*P* < 0.05). Through mediation analysis, it was proven that CAD mediated the causative connection between LDL-C and HCC, and the proportion of mediating effect on HCC was 58.52%. Reverse MR showed that HCC could affect LDL-C levels with a negative correlation (OR_IVW_ = 0.979, 95% CI = [0.961, 0.997], *P* = 0.025).

**Conclusion:**

This MR study confirmed the causal effect between LDL-C levels and HCC risk, with CAD playing a mediating role. It may provide a new view on HCC occurrence and development mechanisms, as well as new metabolic intervention targets for treatment.

**Supplementary Information:**

The online version contains supplementary material available at 10.1186/s12944-023-01877-1.

## Introduction

Liver cancer is one of the most common cancers in the world. Although the global fatality rate of liver cancer has declined slightly in the past decade, it is still at a very high level [1]. It is predicted that by 2025, more than 1 million people will be diagnosed annually with liver cancer [2]. HCC accounts for 80–90% of all primary liver cancers [3]. Risk factors found to be associated with HCC include hepatitis B virus and/or hepatitis C virus infection, nonalcoholic steatohepatitis, excessive alcohol consumption, and family history of HCC [4]. There is growing evidence that metabolic factors are risk factors for HCC. Therefore, identifying the risk factors for HCC and investigating the relationship between risk and protective factors is of utmost importance.

LDL-C is the main pathophysiological factor of atherosclerotic cardiovascular disease [5]. A previous study proved that LDL-C is an important univariate predictor for CAD [6]. The increase in plasma cholesterol may induce HCC [7], but another study found that a relatively low level of LDL is associated with a significant increase in cancer incidence [8]. These contradictory findings show that cholesterol plays a role in HCC development, but the mechanism is not clear. CAD is still the leading cause of death in developed countries [9]. Recent studies have shown that cardiovascular disease (CVD) is associated with the incidence of cancer in patients with CVD [10, 11]. However, the present research evidence indicates that there is no randomized controlled trial (RCT) on CAD and HCC, so we do not know whether there is a potential causal relationship between CAD and HCC.

MR can effectively analyze the causal relationship between exposure and outcomes by utilizing genetic variation to prevent the interference of confounders and reverse causality [12]. Therefore, we conducted a two-sample MR (TSMR) study to explore the causal relationship between LDL-C and HCC, as well as the causal relationship between CAD and HCC. MVMR was used to correct the confounders that might affect causal estimation, and mediation analysis was used to investigate the potential mediating effect of CAD on the causal relationship between LDL-C and HCC. Our study may provide a new perspective for exploring the mechanism of the occurrence and development of HCC and provide novel metabolic therapy intervention targets.

## Method

### Study design

**Fig. 1:**
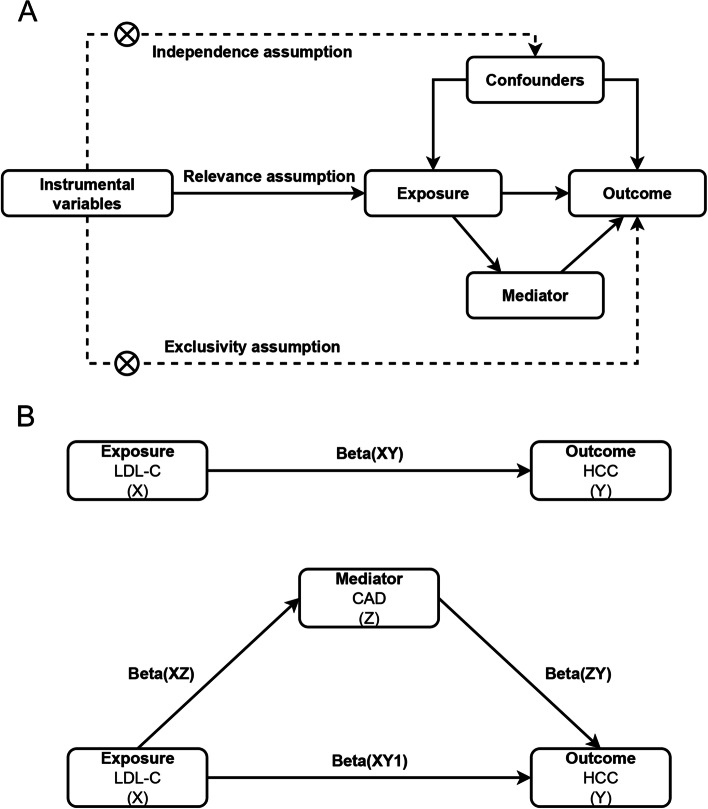
Study design overview. **A** Three critical assumptions of MR analysis. **B** Mediation analysis. LDL-C: Low density lipoprotein cholesterol; CAD: Coronary artery disease; HCC: Hepatocellular carcinoma

We used MR analysis and mediation analysis to identify and estimate the causal relationship between LDL-C and HCC and whether this relationship could be mediated by CAD. As shown in Fig. 1A, the MR analysis has three critical assumptions. We first investigated the overall causative association between LDL-C and HCC and then the ratio of mediating factor CAD to causation (Fig. 1B). This MR study utilized publicly accessible GWAS datasets. In the original GWAS, all participants provided written informed consent.

### Data sources

We downloaded the LDL-C, CAD, and HCC data for our study from BioBank Japan (BBJ), Japanese Encyclopedia of Genetic associations by Riken, the National Bioscience Database Center Human Database and the IEU Open GWAS database. All participants in this study were collected under the BBJ Project. All the data we used were quality controlled, and the following samples were excluded: (i) non–East Asian outliers identified by principal component analysis; (ii) closely related individuals identified by identity-by-descent analysis with Plink2 software; and (iii) sample call rate < 0.98 [13, 14]. Data on LDL-C were obtained from a GWAS conducted by Ishigaki K et al., which included a total of 72,866 individuals [13]. It involved 6,108,953 SNPs and included both males and females. CAD data were available from GWAS of 212,453 individuals (29,319 cases and 183,134 controls) [14]. In addition, the genetic data of HCC were obtained from GWAS including 197,611 samples (1,866 cases and 195,745 controls) [14]. Supplementary Table 1 lists the detailed information of each GWAS summary data in our study.

### Genetic instrumental variable (IV) selection

To obtain SNPs that are significantly linked with LDL-C or CAD, we first set a genome-wide significance threshold of *P* < 5 × 10^–8^. When the number of IVs was insufficient, a relaxed threshold was applied to obtain more IVs connected to exposure, with a maximum threshold of 5 × 10^–6^. Meanwhile, since the existence of linkage disequilibrium (LD) would lead to bias, we set the LD of SNPs significantly related to exposure to satisfy r^2^ < 0.001 and kb > 10,000 [15]. MR-PRESSO was used to detect and correct horizontal pleiotropy by removing outliers [16]. Palindromic SNPs with intermediate allele frequencies were eliminated. In addition, the F-statistic was defined as F = β^2^_exposure_/SE^2^_exposure_ to quantify the genetic tool’s strength for all SNPs, and SNPs with an F value < 10 were considered to be weak instruments [17].

### Mendelian randomization and statistical analysis

TSMR analysis was conducted using R (version 4.2.0) and the package “Two Sample MR”. We predominantly employed the random effects IVW analysis method to determine the causal relationship between exposure and outcome [18]. At the same time, MR‒Egger [19], WM [20], simple mode, and weighted mode [21] were used as auxiliary analysis methods. The impact on the risk of HCC was indicated by OR and 95% CI. In MR analysis, *P* < 0.05 showed that there was a significant causal relationship between exposure and outcome. MVMR was performed using the “MendelianRandomization” and “TwoSampleMR” R packages. IVW was the primary method of analysis, while MR‒Egger was the supplementary method.

### Sensitivity analysis

For sensitivity analysis, we employed the three approaches of heterogeneity test, horizontal pleiotropy test, and leave-one-out method. Cochrane's Q-test was utilized to test for heterogeneity, and a Q *p* value < 0.05 was considered to indicate heterogeneity [22]. If there was heterogeneity, we used IVW with random effects to conduct the study. When the MR‒Egger intercept term was statistically significant, it indicated that there was horizontal pleiotropy. In addition, we used the global test in MR-PRESSO to determine whether there was pleiotropy in this study [16, 23]. To determine the impact of a single SNP on the causal association, "leave-one-out" analysis was used to eliminate each SNP in turn.

### Mediation analysis

The mediating effect was calculated as Beta = Beta(XZ)*Beta(ZY); the proportion of mediating effect in the total effect: R = Beta/Beta(XY)*100%. After correction for confounders, the effect of exposure on outcome was considered to be a direct effect, and direct effect = Beta(XY) – Beta.

### Data visualization

Leave-one-out analysis determined whether a single SNP caused a significant change in the results by eliminating the SNP in turn. Forest plots were employed to assess genetic variation effect estimates. LDL-C, CAD, or HCC, and the comprehensive effect was calculated by IVW. Publication bias was assessed by checking the symmetry of the funnel plots.

## Results

### Selection of IVs

Using thresholds (*P* < 5 × 10^–8^) and removing SNPs with LD, independent SNPs were screened out. Then, the palindrome SNPs were removed, and the outlier SNPs were eliminated by MR-PRESSO analysis. Finally, in the LDL-C versus HCC or CAD analysis, 26 and 22 SNPs were identified as IVs, respectively. Sixty-seven SNPs were chosen as IVs to assess the causal relationship between CAD and HCC. Detailed IV data are shown in Supplementary Table 2.

### Two-sample MR analysis

**Fig. 2:**
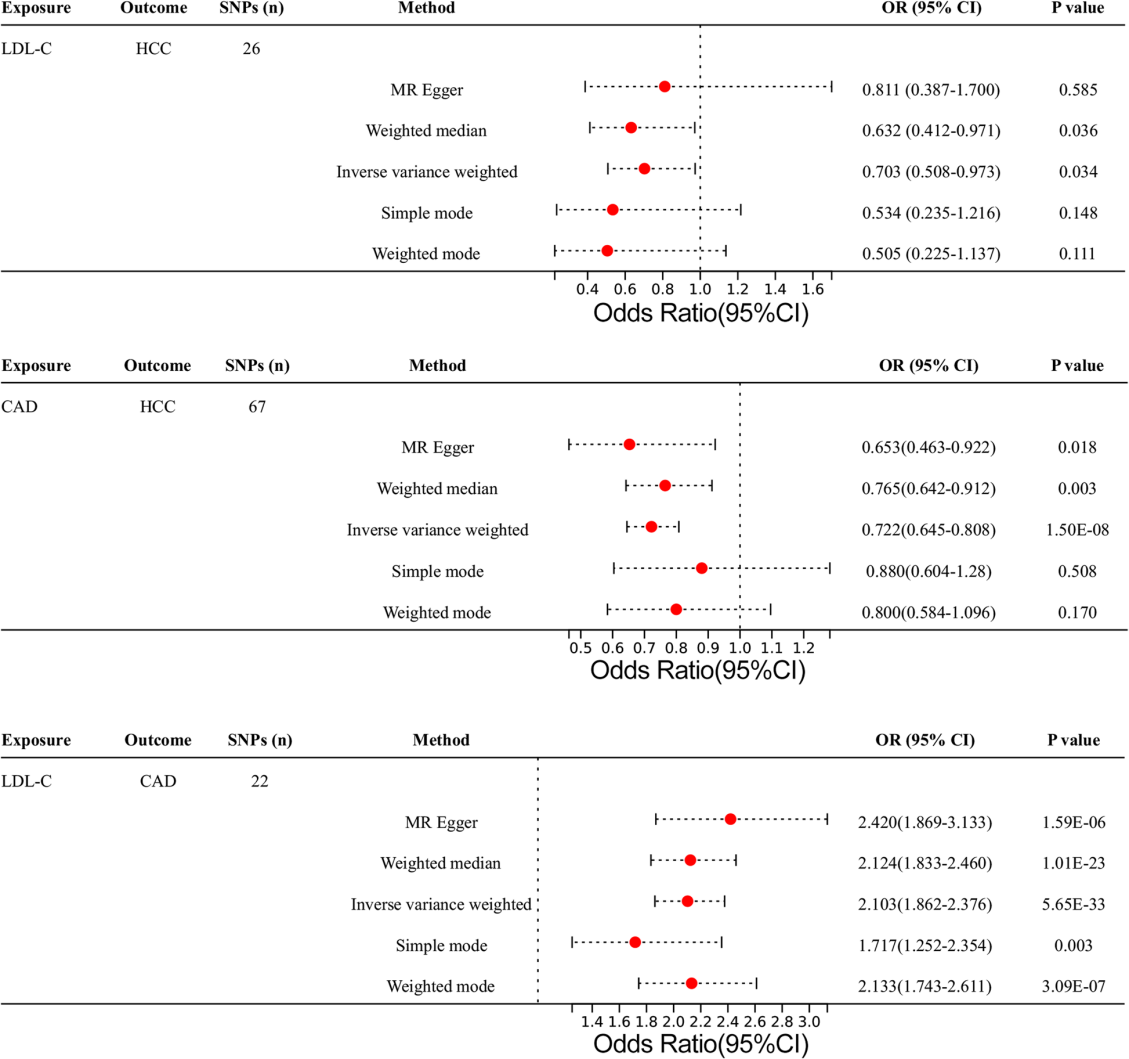
Two-sample MR analysis results. Different MR methods were used to evaluate the causal relationship between LDL-C and CAD and HCC. An OR value greater than 1 indicates that the exposure indicator increases the risk of the outcome; otherwise, it reduces the risk of the outcome

**Fig. 3:**
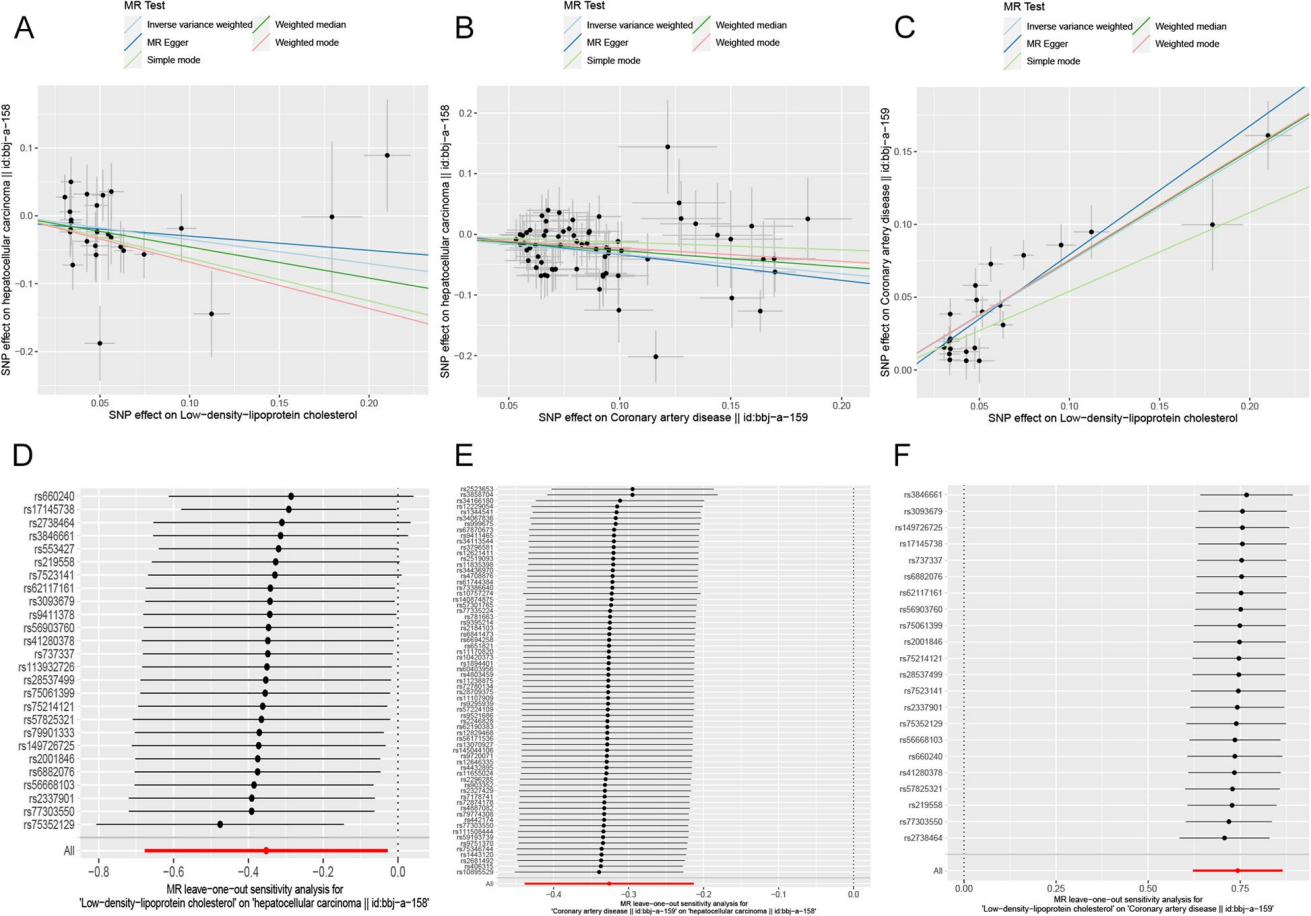
Scatter plots and “leave-one-out” results of genetic correlation between LDL-C, CAD, and HCC by different MR analysis methods. **A**; **D** LDL-C in HCC. **B**; **E** CAD on HCC. **C**; **F** LDL-C on CAD

Figure 2 depicts the MR results from several approaches of analyzing the causal effect of exposure on outcome. The IVW and WM results showed a significant negative causal relationship between LDL-C and HCC (OR_IVW_ = 0.703, 95% CI = [0.508, 0.973], *P* = 0.034; OR_WM_ = 0.632, 95% CI = [0.412, 0.971], *P* = 0.036). IVW, WM, and MR‒Egger analyses all showed a significant causal relationship between CAD and HCC (OR_IVW_ = 0.722, 95% CI = [0.645, 0.808], *P* = 1.50 × 10^–8^; OR_WM_ = 0.765, 95% CI = [0.642, 0.912], *P* = 0.003; OR_MR-Egger_ = 0.653, 95% CI = [0.463, 0.922], *P* = 0.018). Meanwhile, all five analyses showed a significant causal relationship between LDL-C and CAD. Through the trend of fitting results in the scatter chart (Fig. 3A-C), we noticed that as LDL-C increased, the risk of CAD increased, while with the increase in LDL-C or CAD, the risk of HCC decreased.

MR‒Egger and IVW analyses were utilized to detect heterogeneity. MR‒Egger and MR-PRESSO analyses were used to detect horizontal multiplicity. As shown in Table 1, there was no heterogeneity in the MR analysis of LDL-C on HCC and CAD on HCC. However, MR analysis of LDL-C on CAD had heterogeneity, so we used IVW with random effects analysis. In Supplementary Figure S1, the funnel plots for the heterogeneity test are shown. There was no horizontal pleiotropy (Table 1). The result of the “leave-one-out” method showed that the error line did not change much, which means that the MR analysis results were robust (Fig. 3D-F).

**Table 1:** Sensitivity analysis, including heterogeneity test and horizontal pleiotropy test

**Sensitivity analysis**							
**Heterogeneity test**							
**Exposure**	**Outcome**	**Heterogeneity test (MR‒Egger)**			**Heterogeneity test (IVW)**		
		**Cochrane’s Q**	**Q_df**	**Q_pval**	**Cochrane’s Q**	**Q_df**	**Q_pval**
LDL-C	HCC	32.208	24	0.122	32.449	25	0.145
CAD	HCC	72.982	65	0.232	73.389	66	0.249
LDL-C	CAD	39.011	20	0.007	41.834	21	0.004
HCC	LDL-C	4.360	8	0.823	8.494	9	0.485
**Horizontal pleiotropy test**							
**Exposure**	**Outcome**	**Horizontal pleiotropy test (MR‒Egger)**			**Horizontal pleiotropy test (MR-PRESSO)**		
		**Intercept**	**Pval**		**Global test pval**		
LDL-C	HCC	-0.009	0.675		0.293		
CAD	HCC	0.009	0.549		0.276		
LDL-C	CAD	-0.009	0.243		0.196		
HCC	LDL-C	0.014	0.076		0.513		

*LDL-C* Low-Density lipoprotein cholesterol, *CAD* Coronary artery disease, *HCC* Hepatocellular carcinoma, *Q_df* Q_ degree of freedom, *IVW* Inverse variance weighting

### MVMR

A total of 68 IVs were screened out in MVMR analysis (Table S3). As shown in Table 2, after correcting for CAD, the causal effect of LDL-C level on HCC was still significant. Similarly, after correcting for LDL-C level, the causal effect of CAD on the risk of HCC remained significant. There was no heterogeneity in MVMR analysis using the IVW method and MR‒Egger method (IVW: heterogeneity test statistic = 67.4551 on 66 degrees of freedom, *p* value = 0.4271; MR‒Egger: heterogeneity test statistic = 66.6152 on 65 degrees of freedom, *p* value = 0.4212). There was no horizontal pleiotropy through the MR‒Egger (intercept = 0.010, *p* value = 0.365337) and MR-PRESSO (global test *p* value = 0.423) methods. The results of MVMR indicated that LDL-C level and CAD might be jointly involved in the occurrence and development of HCC.

**Table 2:** MVMR analysis of the association between LDL-C/CAD and HCC

Exposure	MV-IVW			MV-Egger		
	Beta	95%CI	Pval	Beta	95%CI	Pval
LDL-C	-0.331	-0.651, -0.012	0.042	-0.380	-0.717, -0.043	0.027
CAD	-0.277	-0.402, -0.151	1.62 × 10^–5^	-0.384	-0.649, -0.119	0.004

*MV-IVW* Multivariate inverse variance weighted, *MV-Egger* Multivariate MR‒Egger

### Mediating effects of CAD on LDL-C-HCC risk

Mediation analysis of CAD was conducted to explore whether the effect of LDL-C on HCC was mediated by it. The findings showed that total effect: Beta(XY) = -0.352. Mediation effect: Beta(XZ)*Beta(ZY) = -0.206. The proportion of mediating effect: R = 58.52%. Direct effect: Beta(XY)-Beta(XZ)*Beta(ZY) = -0.146. The results suggested that CAD may act as a mediator of the causal effect.

### Reverse two-sample MR analysis

In the TSMR analysis between HCC and LDL-C, a relaxed threshold of 5 × 10^–6^ was used to obtain more IVs. A total of 10 SNPs were identified. The IVW results showed a significant negative causal relationship between HCC and LDL-C (OR_IVW_ = 0.979, 95% CI = [0.961, 0.997], *P* = 0.025). The same result was observed in the MR‒Egger method (*P* = 0.032). However, the weighted median method showed a lack of significant correlation (*P* > 0.05). In the MR analysis of HCC on LDL-C, neither heterogeneity nor horizontal pleiotropy was present (Table 1). However, the results of TSMR analysis showed that there was no causal relationship between CAD as exposure and LDL-C levels as an outcome (*P* > 0.05).

## Discussion

Based on the results of MR analysis, our study revealed that there is a causal relationship between LDL-C level and HCC in the East Asian population. MVMR and mediation analysis also emphasized the mediating role of CAD in the causal association between LDL-C and HCC. This may provide a new perspective on the mechanism of the occurrence and development of HCC and provide a new metabolic intervention target for treatment.

HCC is one of the leading cancers in the world. The main risk factors for HCC include alcohol consumption, nonalcoholic fatty liver disease, and HBV or HCV infection [24]. Recently, there has been increasing evidence that metabolic factors, including dyslipidemia and metabolic syndrome, are risk factors for HCC [25–27]. Most previous studies have shown that dyslipidemia is one of the major risk factors for CAD. LDL is the lipoprotein with the highest cholesterol content in plasma and is the main component of the lipid core of atherosclerotic plaques. There is a great deal of evidence that LDL is the main pathogenic factor in the occurrence and development of CAD. With the increase in LDL level, the risk of CAD increases [5, 28]. By reducing the level of LDL, the relative risk of CAD can be reduced [29]. LDL-C is a recognized indicator of LDL. There are significant levels of small dense LDL (sdLDL) in the blood of patients with acute coronary syndrome [30]. The increase in serum sdLDL is related to the occurrence and development of CAD [31]. In addition, oxidized LDL (ox-LDL) is the main risk factor for atherosclerosis, as demonstrated by numerous studies [32]. A prospective study of the Chinese population found that a relatively low level of LDL-C (< 100 mg/dl) was associated with a significant increase in the incidence of cancer [1.20 (1.08–1.34); *P* = 0.0007] [8]. A study by Dong Hyun Sinn et al. found that hypercholesterolemia is associated with a lower risk of HCC [33]. In addition, a study of the Korean population showed that with the increase in total cholesterol and LDL-C, the incidence of HCC gradually decreased. Obviously, this conclusion is contrary to common sense in the past, but it is consistent with the conclusion of our MR research. In this study, through TSMR, we found that there is a significant negative causal relationship between LDL-C and HCC. With the increase in LDL-C levels, the risk of HCC decreases. At the same time, reverse TSMR results prove that there is a causal relationship between the risk of HCC and the level of LDL-C. However, the causal relationship between CAD and HCC has received scant attention. Therefore, we use TSMR to analyze the causality between CAD and HCC and MVMR and mediation analysis to determine that CAD has a mediating effect between LDL-C and HCC, which is robust and consistent in sensitivity analysis.

### Study strengths and limitations

MR, the major advantage of this study, used single nucleotide polymorphisms as instrumental variables to analyze the relationship between exposure and outcome. Compared with RCT, MR reduces the bias caused by confounders and prevents the interference of reverse causality. We utilized TSMR to study the linear link between exposure and outcome, as well as MVMR and mediation analysis to examine potential nonlinear correlations. In addition, the data we used were all from East Asian population samples, which substantially decreased population heterogeneity bias. Finally, we conducted several sensitivity analyses in this study to ensure that the results were robust and reliable. However, this study has several limitations that need to be considered. First, our study focused on East Asian populations, so further research is needed to extend our findings to other ethnic groups. Second, the association between LDL-C and HCC is mediated by many factors, and our study cannot completely avoid the interference of confounders. Third, the HCC data we used were from public databases, and we were unable to conduct a subgroup analysis of specific factors, such as sex and age.

## Conclusions

In conclusion, through MR analysis, this study presented genetic evidence of a causal relationship between LDL-C and HCC. That is, the higher the LDL-C level, the lower the risk of HCC, with CAD serving as a mediator. This may provide new insights into the mechanism of the occurrence and development of HCC and provide new metabolic intervention targets for treatment.

## Supplementary Information

**Additional file 1.**

## Data Availability

The datasets generated and/or analyzed during the current study are available in BioBank Japan (https://biobankjp.org/en/), the Japanese Encyclopedia of Genetic associations by Riken (http://jenger.riken.jp/en/), the National Bioscience Database Center Human Database (https://humandbs.biosciencedbc.jp/en/) (research ID: hum0014) and the IEU OPEN GWAS PROJECT repository (https://gwas.mrcieu.ac.uk/).

## Abbreviations

LDL-C: Low-density lipoprotein cholesterol
HCC: Hepatocellular carcinoma
MR: Mendelian randomization
CAD: Coronary artery disease
GWAS: Genome-wide association studies
IVW: Inverse variance weighting
WM: Weighted median
MVMR: Multivariate Mendelian randomization
OR: Odds ratio
CI: Confidence interval
CVD: Cardiovascular disease
RCT: Randomized controlled trial
TSMR: Two-sample Mendelian randomization
BBJ: BioBank Japan
LD: Linkage disequilibrium
sdLDL: Small dense LDL
ox-LDL: Oxidized LDL
