# Supplementary material for: Low-density lipoprotein cholesterol and risk of hepatocellular carcinoma: a Mendelian randomization and mediation analysis
Source: Lipids Health Dis. 2023 Jul 31;22:110. doi: 10.1186/s12944-023-01877-1 (PMC10388495; doi:10.1186/s12944-023-01877-1)
Supplement: Supplementary file 1 — Additional file 1. [file 12944_2023_1877_MOESM1_ESM.docx]

| **Phenotype** | **GWAS ID** | **Sample Size** | **N case** | **N control** | **Population** | **N SNPs** | **PMID** |  |
| --- | --- | --- | --- | --- | --- | --- | --- | --- |
| **Low-density-lipoprotein cholesterol** | bbj-a-31 | 72,866 | - | - | East Asian | 6,108,953 | 29403010 |  |
| **Coronary artery disease** | bbj-a-159 | 212,453 | 29,319 | 183,134 | East Asian | 8,881,048 | 32514122 |  |
| **Hepatocellular carcinoma** | bbj-a-158 | 197,611 | 1,866 | 195,745 | East Asian | 8,885,115 | 32514122 |  |

**Supplementary Table 1** Detailed information of data sources for analyses.

**Supplementary Table 2** Information on instrumental variables of LDL-C, CAD and HCC.

**1. LDL-C – HCC**

| **SNP** | **Chr** | **EA** | **OA** | **Beta** | **SE** | **Pval** | **Eaf** | **F** |
| --- | --- | --- | --- | --- | --- | --- | --- | --- |
| rs113932726 | 11 | T | C | -0.05462 | 0.009683 | 1.70E-08 | 0.0754 | 31.81878 |
| rs149726725 | 1 | G | A | 0.1793 | 0.01705 | 7.11E-26 | 0.0265 | 110.589 |
| rs17145738 | 7 | T | C | 0.04998 | 0.008538 | 4.83E-09 | 0.1038 | 34.26732 |
| rs2001846 | 8 | C | T | -0.03046 | 0.005245 | 6.34E-09 | 0.5657 | 33.72631 |
| rs219558 | 2 | T | G | 0.04785 | 0.006992 | 7.76E-12 | 0.2107 | 46.83398 |
| rs2337901 | 2 | T | C | -0.05166 | 0.005941 | 3.43E-18 | 0.744 | 75.61182 |
| rs2738464 | 19 | C | G | 0.07458 | 0.005624 | 3.84E-40 | 0.685 | 175.8548 |
| rs28537499 | 7 | C | A | -0.03394 | 0.005325 | 1.84E-10 | 0.391 | 40.62417 |
| rs3093679 | 17 | A | G | -0.04275 | 0.00712 | 1.92E-09 | 0.1565 | 36.05058 |
| rs3846661 | 5 | G | A | 0.06316 | 0.005451 | 4.70E-31 | 0.5107 | 134.2554 |
| rs41280378 | 10 | G | T | -0.04845 | 0.006252 | 9.19E-15 | 0.2488 | 60.05506 |
| rs553427 | 1 | T | C | 0.03482 | 0.005757 | 1.46E-09 | 0.7098 | 36.5818 |
| rs56668103 | 20 | C | T | 0.03384 | 0.00585 | 7.27E-09 | 0.7229 | 33.46178 |
| rs56903760 | 15 | T | C | 0.0334 | 0.005917 | 1.65E-08 | 0.2705 | 31.86323 |
| rs57825321 | 2 | A | T | -0.09537 | 0.008365 | 4.11E-30 | 0.1265 | 129.9845 |
| rs62117161 | 19 | G | A | 0.04745 | 0.007993 | 2.93E-09 | 0.131 | 35.24137 |
| rs660240 | 1 | C | T | 0.1122 | 0.01013 | 1.79E-28 | 0.9282 | 122.678 |
| rs6882076 | 5 | C | T | 0.04276 | 0.007473 | 1.05E-08 | 0.8232 | 32.74051 |
| rs737337 | 19 | C | T | -0.03363 | 0.005887 | 1.11E-08 | 0.2662 | 32.63365 |
| rs75061399 | 12 | A | G | 0.03396 | 0.006221 | 4.80E-08 | 0.264 | 29.79991 |
| rs75214121 | 8 | T | C | -0.03326 | 0.005784 | 8.88E-09 | 0.2812 | 33.06648 |
| rs7523141 | 1 | T | C | 0.06155 | 0.00592 | 2.55E-25 | 0.7124 | 108.0968 |
| rs75352129 | 2 | T | C | 0.2101 | 0.01298 | 5.64E-59 | 0.0522 | 262.0009 |
| rs77303550 | 16 | T | C | -0.05635 | 0.00698 | 6.85E-16 | 0.2326 | 65.17439 |
| rs79901333 | 1 | G | A | 0.04827 | 0.006763 | 9.52E-13 | 0.1858 | 50.94198 |
| rs9411378 | 9 | A | C | 0.05624 | 0.006199 | 1.16E-19 | 0.2562 | 82.309 |

**2. LDL-C – CAD**

| **SNP** | **Chr** | **EA** | **OA** | **Beta** | **SE** | **Pval** | **Eaf** | **F** |
| --- | --- | --- | --- | --- | --- | --- | --- | --- |
| rs149726725 | 1 | G | A | 0.1793 | 0.01705 | 7.11E-26 | 0.0265 | 110.589 |
| rs17145738 | 7 | T | C | 0.04998 | 0.008538 | 4.83E-09 | 0.1038 | 34.26732 |
| rs2001846 | 8 | C | T | -0.03046 | 0.005245 | 6.34E-09 | 0.5657 | 33.72631 |
| rs219558 | 2 | T | G | 0.04785 | 0.006992 | 7.76E-12 | 0.2107 | 46.83398 |
| rs2337901 | 2 | T | C | -0.05166 | 0.005941 | 3.43E-18 | 0.744 | 75.61182 |
| rs2738464 | 19 | C | G | 0.07458 | 0.005624 | 3.84E-40 | 0.685 | 175.8548 |
| rs28537499 | 7 | C | A | -0.03394 | 0.005325 | 1.84E-10 | 0.391 | 40.62417 |
| rs3093679 | 17 | A | G | -0.04275 | 0.00712 | 1.92E-09 | 0.1565 | 36.05058 |
| rs3846661 | 5 | G | A | 0.06316 | 0.005451 | 4.7E-31 | 0.5107 | 134.2554 |
| rs41280378 | 10 | G | T | -0.04845 | 0.006252 | 9.19E-15 | 0.2488 | 60.05506 |
| rs56668103 | 20 | C | T | 0.03384 | 0.00585 | 7.27E-09 | 0.7229 | 33.46178 |
| rs56903760 | 15 | T | C | 0.0334 | 0.005917 | 1.65E-08 | 0.2705 | 31.86323 |
| rs57825321 | 2 | A | T | -0.09537 | 0.008365 | 4.11E-30 | 0.1265 | 129.9845 |
| rs62117161 | 19 | G | A | 0.04745 | 0.007993 | 2.93E-09 | 0.131 | 35.24137 |
| rs660240 | 1 | C | T | 0.1122 | 0.01013 | 1.79E-28 | 0.9282 | 122.678 |
| rs6882076 | 5 | C | T | 0.04276 | 0.007473 | 1.05E-08 | 0.8232 | 32.74051 |
| rs737337 | 19 | C | T | -0.03363 | 0.005887 | 1.11E-08 | 0.2662 | 32.63365 |
| rs75061399 | 12 | A | G | 0.03396 | 0.006221 | 4.8E-08 | 0.264 | 29.79991 |
| rs75214121 | 8 | T | C | -0.03326 | 0.005784 | 8.88E-09 | 0.2812 | 33.06648 |
| rs7523141 | 1 | T | C | 0.06155 | 0.00592 | 2.55E-25 | 0.7124 | 108.0968 |
| rs75352129 | 2 | T | C | 0.2101 | 0.01298 | 5.64E-59 | 0.0522 | 262.0009 |
| rs77303550 | 16 | T | C | -0.05635 | 0.00698 | 6.85E-16 | 0.2326 | 65.17439 |

**3. CAD - HCC**

| **SNP** | **Chr** | **EA** | **OA** | **Beta** | **SE** | **Pval** | **Eaf** | **F** |
| --- | --- | --- | --- | --- | --- | --- | --- | --- |
| rs10420373 | 19 | T | G | -0.0569 | 0.010076 | 1.63E-08 | 0.452549 | 31.89506 |
| rs10757274 | 9 | G | A | 0.169942 | 0.009448 | 2.43E-72 | 0.475325 | 323.56 |
| rs10895529 | 11 | G | C | -0.09069 | 0.00991 | 5.64E-20 | 0.367605 | 83.74022 |
| rs11107909 | 12 | T | C | -0.09402 | 0.011343 | 1.14E-16 | 0.232879 | 68.70681 |
| rs111508444 | 6 | G | A | 0.159433 | 0.018738 | 1.76E-17 | 0.06932 | 72.3991 |
| rs11170820 | 12 | G | C | 0.089349 | 0.014913 | 2.08E-09 | 0.1519 | 35.89876 |
| rs11238875 | 10 | G | A | -0.05329 | 0.009621 | 3.03E-08 | 0.542468 | 30.68528 |
| rs11655024 | 17 | C | T | -0.08637 | 0.013532 | 1.74E-10 | 0.834191 | 40.7336 |
| rs11835398 | 12 | G | C | 0.062351 | 0.010002 | 4.55E-10 | 0.361396 | 38.86158 |
| rs12229054 | 12 | A | G | 0.150483 | 0.014443 | 2.03E-25 | 0.162057 | 108.5591 |
| rs12621411 | 2 | A | C | 0.070056 | 0.01041 | 1.7E-11 | 0.340682 | 45.28886 |
| rs12646335 | 4 | A | G | -0.05803 | 0.009559 | 1.27E-09 | 0.461238 | 36.85756 |
| rs12829468 | 12 | C | A | 0.055099 | 0.009527 | 7.33E-09 | 0.487282 | 33.4456 |
| rs13070927 | 3 | T | G | -0.07234 | 0.011543 | 3.68E-10 | 0.213807 | 39.27629 |
| rs1344541 | 12 | A | G | 0.099592 | 0.015492 | 1.29E-10 | 0.101993 | 41.32766 |
| rs140874875 | 17 | C | CTT | 0.058777 | 0.010518 | 2.29E-08 | 0.70545 | 31.22999 |
| rs1443120 | 11 | G | C | 0.078821 | 0.009701 | 4.47E-16 | 0.413025 | 66.01618 |
| rs145044106 | 6 | C | G | 0.150017 | 0.023545 | 1.87E-10 | 0.041673 | 40.59603 |
| rs1894401 | 15 | A | G | -0.10077 | 0.013811 | 2.97E-13 | 0.821821 | 53.22953 |
| rs2184103 | 1 | G | A | 0.059524 | 0.009571 | 4.99E-10 | 0.491388 | 38.6793 |
| rs2246828 | 10 | A | G | 0.065896 | 0.01135 | 6.41E-09 | 0.227854 | 33.7064 |
| rs2296285 | 13 | A | T | 0.079307 | 0.009878 | 9.88E-16 | 0.384034 | 64.45416 |
| rs2327429 | 6 | C | T | -0.0992 | 0.00946 | 9.98E-26 | 0.517734 | 109.9628 |
| rs2519093 | 9 | T | C | 0.06952 | 0.010587 | 5.16E-11 | 0.273596 | 43.11718 |
| rs2523653 | 6 | G | A | -0.11624 | 0.012461 | 1.08E-20 | 0.800863 | 87.01756 |
| rs2681492 | 12 | C | T | 0.067722 | 0.009767 | 4.09E-12 | 0.377659 | 48.07919 |
| rs28709375 | 1 | G | A | 0.095031 | 0.009483 | 1.22E-23 | 0.542939 | 100.4333 |
| rs34067836 | 12 | CCT | C | -0.09381 | 0.0096 | 1.48E-22 | 0.526506 | 95.4987 |
| rs34113544 | 19 | CA | C | 0.080621 | 0.010037 | 9.57E-16 | 0.665469 | 64.51704 |
| rs34166180 | 15 | A | C | -0.09088 | 0.009777 | 1.47E-20 | 0.551859 | 86.40375 |
| rs34436970 | 2 | A | G | 0.099307 | 0.013845 | 7.36E-13 | 0.134378 | 51.44694 |
| rs3796581 | 4 | G | A | -0.06455 | 0.011209 | 8.5E-09 | 0.233663 | 33.15829 |
| rs3858704 | 12 | G | A | -0.16331 | 0.010142 | 2.45E-58 | 0.677751 | 259.2901 |
| rs406315 | 19 | A | G | 0.121618 | 0.022275 | 4.76E-08 | 0.941437 | 29.81122 |
| rs442174 | 6 | A | T | 0.127648 | 0.018114 | 1.83E-12 | 0.072378 | 49.6586 |
| rs4432895 | 5 | T | C | 0.059597 | 0.010429 | 1.1E-08 | 0.683696 | 32.65625 |
| rs4708876 | 6 | G | C | -0.09249 | 0.014287 | 9.55E-11 | 0.873705 | 41.91178 |
| rs4803459 | 19 | C | T | -0.07323 | 0.009704 | 4.48E-14 | 0.487713 | 56.94499 |
| rs4887082 | 15 | C | T | -0.08607 | 0.0099 | 3.49E-18 | 0.442067 | 75.59101 |
| rs56171536 | 6 | C | A | 0.14403 | 0.024854 | 6.83E-09 | 0.046533 | 33.5836 |
| rs57224109 | 6 | G | A | 0.169562 | 0.018526 | 5.56E-20 | 0.069718 | 83.7701 |
| rs57301765 | 7 | A | G | 0.063197 | 0.01014 | 4.6E-10 | 0.346948 | 38.842 |
| rs59193739 | 6 | T | C | 0.134208 | 0.016032 | 5.7E-17 | 0.095513 | 70.07708 |
| rs60403956 | 2 | G | A | -0.08524 | 0.01551 | 3.89E-08 | 0.133426 | 30.20555 |
| rs61744384 | 11 | A | T | 0.064741 | 0.009818 | 4.28E-11 | 0.384325 | 43.48139 |
| rs62190383 | 2 | A | G | -0.05624 | 0.009448 | 2.65E-09 | 0.489612 | 35.42841 |
| rs651821 | 11 | T | C | -0.06199 | 0.010164 | 1.07E-09 | 0.651116 | 37.18992 |
| rs6694258 | 1 | A | C | -0.0552 | 0.009503 | 6.32E-09 | 0.495844 | 33.73217 |
| rs67870673 | 1 | T | C | -0.07106 | 0.009627 | 1.57E-13 | 0.424487 | 54.48645 |
| rs6841473 | 4 | T | C | 0.094958 | 0.010199 | 1.27E-20 | 0.313352 | 86.68428 |
| rs7178741 | 15 | A | C | 0.0668 | 0.010813 | 6.5E-10 | 0.391274 | 38.16554 |
| rs72780134 | 2 | A | G | -0.08066 | 0.013896 | 6.45E-09 | 0.137936 | 33.69417 |
| rs72874178 | 2 | A | G | -0.07718 | 0.010032 | 1.43E-14 | 0.362166 | 59.18877 |
| rs73386640 | 11 | G | A | 0.092428 | 0.014296 | 1.01E-10 | 0.12433 | 41.80066 |
| rs75346744 | 2 | G | A | 0.18474 | 0.01983 | 1.21E-20 | 0.061467 | 86.7896 |
| rs77303550 | 16 | T | C | -0.07275 | 0.012067 | 1.65E-09 | 0.214902 | 36.34379 |
| rs77335224 | 10 | T | C | -0.09334 | 0.011461 | 3.81E-16 | 0.238781 | 66.32994 |
| rs781663 | 4 | G | A | 0.058117 | 0.009753 | 2.54E-09 | 0.389744 | 35.50609 |
| rs79774308 | 6 | G | A | 0.126726 | 0.021071 | 1.81E-09 | 0.054899 | 36.17174 |
| rs903352 | 13 | C | A | -0.07459 | 0.009717 | 1.63E-14 | 0.618829 | 58.93318 |
| rs9295939 | 6 | G | A | 0.164702 | 0.018364 | 3E-19 | 0.070084 | 80.44011 |
| rs9395214 | 6 | G | C | 0.112544 | 0.012234 | 3.6E-20 | 0.802938 | 84.62953 |
| rs9411465 | 9 | A | G | 0.066237 | 0.010863 | 1.08E-09 | 0.268052 | 37.17718 |
| rs9521686 | 13 | C | T | 0.082908 | 0.010213 | 4.75E-16 | 0.690853 | 65.89673 |
| rs9720071 | 7 | C | A | -0.06674 | 0.011898 | 2.03E-08 | 0.270387 | 31.46805 |
| rs9751370 | 2 | G | A | 0.064917 | 0.010002 | 8.56E-11 | 0.498724 | 42.12571 |
| rs999675 | 16 | T | C | 0.067326 | 0.009509 | 1.44E-12 | 0.551686 | 50.13437 |

**4. HCC – LDL-C**

| **SNP** | **Chr** | **EA** | **OA** | **Beta** | **SE** | **Pval** | **Eaf** | **F** |
| --- | --- | --- | --- | --- | --- | --- | --- | --- |
| rs113721058 | 1 | A | G | 0.320495 | 0.061606 | 1.97E-07 | 0.084839 | 27.06419 |
| rs113777417 | 6 | C | A | 0.203222 | 0.034047 | 2.39E-09 | 0.589394 | 35.62653 |
| rs12901503 | 15 | G | A | -0.1647 | 0.034283 | 1.56E-06 | 0.394697 | 23.07883 |
| rs1453025 | 5 | G | A | 0.164545 | 0.033584 | 9.61E-07 | 0.518916 | 24.00516 |
| rs3747207 | 22 | A | G | 0.165852 | 0.033293 | 6.31E-07 | 0.455823 | 24.81651 |
| rs377743 | 6 | A | G | 0.180623 | 0.034534 | 1.69E-07 | 0.430597 | 27.35583 |
| rs401556 | 6 | A | G | -0.21616 | 0.045828 | 2.40E-06 | 0.154741 | 22.24789 |
| rs61827280 | 1 | T | C | 0.331098 | 0.064116 | 2.42E-07 | 0.07951 | 26.66746 |
| rs72501964 | 4 | T | G | 0.168088 | 0.03567 | 2.45E-06 | 0.349477 | 22.20559 |
| rs8107030 | 19 | G | A | 0.367335 | 0.05977 | 7.96E-10 | 0.100965 | 37.77109 |

**5. CAD – LDL-C**

| **SNP** | **Chr** | **EA** | **OA** | **Beta** | **SE** | **Pval** | **Eaf** | **F** |
| --- | --- | --- | --- | --- | --- | --- | --- | --- |
| rs10420373 | 19 | T | G | -0.0569 | 0.010076 | 1.63E-08 | 0.452549 | 31.89506 |
| rs10895529 | 11 | G | C | -0.09069 | 0.00991 | 5.64E-20 | 0.367605 | 83.74022 |
| rs11107909 | 12 | T | C | -0.09402 | 0.011343 | 1.14E-16 | 0.232879 | 68.70681 |
| rs11170820 | 12 | G | C | 0.089349 | 0.014913 | 2.08E-09 | 0.1519 | 35.89876 |
| rs11238875 | 10 | G | A | -0.05329 | 0.009621 | 3.03E-08 | 0.542468 | 30.68528 |
| rs11655024 | 17 | C | T | -0.08637 | 0.013532 | 1.74E-10 | 0.834191 | 40.7336 |
| rs11835398 | 12 | G | C | 0.062351 | 0.010002 | 4.55E-10 | 0.361396 | 38.86158 |
| rs12646335 | 4 | A | G | -0.05803 | 0.009559 | 1.27E-09 | 0.461238 | 36.85756 |
| rs12829468 | 12 | C | A | 0.055099 | 0.009527 | 7.33E-09 | 0.487282 | 33.4456 |
| rs13070927 | 3 | T | G | -0.07234 | 0.011543 | 3.68E-10 | 0.213807 | 39.27629 |
| rs1344541 | 12 | A | G | 0.099592 | 0.015492 | 1.29E-10 | 0.101993 | 41.32766 |
| rs1443120 | 11 | G | C | 0.078821 | 0.009701 | 4.47E-16 | 0.413025 | 66.01618 |
| rs145044106 | 6 | C | G | 0.150017 | 0.023545 | 1.87E-10 | 0.041673 | 40.59603 |
| rs1894401 | 15 | A | G | -0.10077 | 0.013811 | 2.97E-13 | 0.821821 | 53.22953 |
| rs2184103 | 1 | G | A | 0.059524 | 0.009571 | 4.99E-10 | 0.491388 | 38.6793 |
| rs2246828 | 10 | A | G | 0.065896 | 0.01135 | 6.41E-09 | 0.227854 | 33.7064 |
| rs2296285 | 13 | A | T | 0.079307 | 0.009878 | 9.88E-16 | 0.384034 | 64.45416 |
| rs2327429 | 6 | C | T | -0.0992 | 0.00946 | 9.98E-26 | 0.517734 | 109.9628 |
| rs2681492 | 12 | C | T | 0.067722 | 0.009767 | 4.09E-12 | 0.377659 | 48.07919 |
| rs28709375 | 1 | G | A | 0.095031 | 0.009483 | 1.22E-23 | 0.542939 | 100.4333 |
| rs34166180 | 15 | A | C | -0.09088 | 0.009777 | 1.47E-20 | 0.551859 | 86.40375 |
| rs3796581 | 4 | G | A | -0.06455 | 0.011209 | 8.50E-09 | 0.233663 | 33.15829 |
| rs4359307 | 13 | C | G | -0.06182 | 0.010166 | 1.19E-09 | 0.566496 | 36.97978 |
| rs442174 | 6 | A | T | 0.127648 | 0.018114 | 1.83E-12 | 0.072378 | 49.6586 |
| rs4432895 | 5 | T | C | 0.059597 | 0.010429 | 1.10E-08 | 0.683696 | 32.65625 |
| rs4708876 | 6 | G | C | -0.09249 | 0.014287 | 9.55E-11 | 0.873705 | 41.91178 |
| rs4803459 | 19 | C | T | -0.07323 | 0.009704 | 4.48E-14 | 0.487713 | 56.94499 |
| rs56171536 | 6 | C | A | 0.14403 | 0.024854 | 6.83E-09 | 0.046533 | 33.5836 |
| rs57224109 | 6 | G | A | 0.169562 | 0.018526 | 5.56E-20 | 0.069718 | 83.7701 |
| rs57301765 | 7 | A | G | 0.063197 | 0.01014 | 4.60E-10 | 0.346948 | 38.842 |
| rs59193739 | 6 | T | C | 0.134208 | 0.016032 | 5.70E-17 | 0.095513 | 70.07708 |
| rs61744384 | 11 | A | T | 0.064741 | 0.009818 | 4.28E-11 | 0.384325 | 43.48139 |
| rs62190383 | 2 | A | G | -0.05624 | 0.009448 | 2.65E-09 | 0.489612 | 35.42841 |
| rs6694258 | 1 | A | C | -0.0552 | 0.009503 | 6.32E-09 | 0.495844 | 33.73217 |
| rs67870673 | 1 | T | C | -0.07106 | 0.009627 | 1.57E-13 | 0.424487 | 54.48645 |
| rs6841473 | 4 | T | C | 0.094958 | 0.010199 | 1.27E-20 | 0.313352 | 86.68428 |
| rs72874178 | 2 | A | G | -0.07718 | 0.010032 | 1.43E-14 | 0.362166 | 59.18877 |
| rs73386640 | 11 | G | A | 0.092428 | 0.014296 | 1.01E-10 | 0.12433 | 41.80066 |
| rs77335224 | 10 | T | C | -0.09334 | 0.011461 | 3.81E-16 | 0.238781 | 66.32994 |
| rs781663 | 4 | G | A | 0.058117 | 0.009753 | 2.54E-09 | 0.389744 | 35.50609 |
| rs79774308 | 6 | G | A | 0.126726 | 0.021071 | 1.81E-09 | 0.054899 | 36.17174 |
| rs903352 | 13 | C | A | -0.07459 | 0.009717 | 1.63E-14 | 0.618829 | 58.93318 |
| rs9295939 | 6 | G | A | 0.164702 | 0.018364 | 3.00E-19 | 0.070084 | 80.44011 |
| rs9395214 | 6 | G | C | 0.112544 | 0.012234 | 3.60E-20 | 0.802938 | 84.62953 |
| rs9521686 | 13 | C | T | 0.082908 | 0.010213 | 4.75E-16 | 0.690853 | 65.89673 |
| rs9751370 | 2 | G | A | 0.064917 | 0.010002 | 8.56E-11 | 0.498724 | 42.12571 |
| rs999675 | 16 | T | C | 0.067326 | 0.009509 | 1.44E-12 | 0.551686 | 50.13437 |

SNP: single nucleotide polymorphism; Chr: chromosome; EA: effect allele; OA: other allele; Beta was obtained by allele-related effects; SE: standard error; Eaf: effect allele frequency. Beta, SE, and Pval are SNP summary statistics; LDL-C: Low-density-lipoprotein cholesterol; HCC: Hepatocellular carcinoma; CAD: Coronary artery disease.

**Supplementary Table 3** IVs in MVMR analysis.

**Instrumental Variable**

**LDL-C and CAD – HCC**

| **Exposure** | **SNP** | **EA** | **OA** | **Beta** | **SE** | **Pval** | **Eaf** | **F** |
| --- | --- | --- | --- | --- | --- | --- | --- | --- |
| **LDL-C** | rs56903760 | T | C | 0.0334 | 0.005917 | 1.65E-08 | 0.2705 | 31.86323 |
|  | rs737337 | C | T | -0.03363 | 0.005887 | 1.11E-08 | 0.2662 | 32.63365 |
|  | rs6882076 | C | T | 0.04276 | 0.007473 | 1.05E-08 | 0.8232 | 32.74051 |
|  | rs56668103 | C | T | 0.03384 | 0.00585 | 7.27E-09 | 0.7229 | 33.46178 |
|  | rs2001846 | C | T | -0.03046 | 0.005245 | 6.34E-09 | 0.5657 | 33.72631 |
|  | rs17145738 | T | C | 0.04998 | 0.008538 | 4.83E-09 | 0.1038 | 34.26732 |
|  | rs3093679 | A | G | -0.04275 | 0.00712 | 1.92E-09 | 0.1565 | 36.05058 |
|  | rs553427 | T | C | 0.03482 | 0.005757 | 1.46E-09 | 0.7098 | 36.5818 |
|  | rs28537499 | C | A | -0.03394 | 0.005325 | 1.84E-10 | 0.391 | 40.62417 |
|  | rs79901333 | G | A | 0.04827 | 0.006763 | 9.52E-13 | 0.1858 | 50.94198 |
|  | rs3858704 | G | A | -0.04119 | 0.005519 | 8.4E-14 | 0.6775 | 55.70097 |
|  | rs9411465 | A | G | 0.04545 | 0.006082 | 7.84E-14 | 0.2744 | 55.8438 |
|  | rs41280378 | G | T | -0.04845 | 0.006252 | 9.19E-15 | 0.2488 | 60.05506 |
|  | rs77303550 | T | C | -0.05635 | 0.00698 | 6.85E-16 | 0.2326 | 65.17439 |
|  | rs34436970 | A | G | 0.06194 | 0.007491 | 1.36E-16 | 0.1348 | 68.36956 |
|  | rs9411378 | A | C | 0.05624 | 0.006199 | 1.16E-19 | 0.2562 | 82.309 |
|  | rs7523141 | T | C | 0.06155 | 0.00592 | 2.55E-25 | 0.7124 | 108.0968 |
|  | rs72780134 | A | G | -0.08315 | 0.007985 | 2.16E-25 | 0.1296 | 108.4363 |
|  | rs660240 | C | T | 0.1122 | 0.01013 | 1.79E-28 | 0.9282 | 122.678 |
|  | rs3846661 | G | A | 0.06316 | 0.005451 | 4.7E-31 | 0.5107 | 134.2554 |
|  | rs2738464 | C | G | 0.07458 | 0.005624 | 3.84E-40 | 0.685 | 175.8548 |
|  | rs75346744 | G | A | 0.1612 | 0.01056 | 1.41E-52 | 0.0611 | 233.0249 |
| **CAD** | rs11238875 | G | A | -0.05329 | 0.009621 | 3.03E-08 | 0.542468 | 30.68528 |
|  | rs10420373 | T | G | -0.0569 | 0.010076 | 1.63E-08 | 0.452549 | 31.89506 |
|  | rs4432895 | T | C | 0.059597 | 0.010429 | 1.1E-08 | 0.683696 | 32.65625 |
|  | rs3796581 | G | A | -0.06455 | 0.011209 | 8.5E-09 | 0.233663 | 33.15829 |
|  | rs12829468 | C | A | 0.055099 | 0.009527 | 7.33E-09 | 0.487282 | 33.4456 |
|  | rs56171536 | C | A | 0.14403 | 0.024854 | 6.83E-09 | 0.046533 | 33.5836 |
|  | rs72780134 | A | G | -0.08066 | 0.013896 | 6.45E-09 | 0.137936 | 33.69417 |
|  | rs2246828 | A | G | 0.065896 | 0.01135 | 6.41E-09 | 0.227854 | 33.7064 |
|  | rs6694258 | A | C | -0.0552 | 0.009503 | 6.32E-09 | 0.495844 | 33.73217 |
|  | rs62190383 | A | G | -0.05624 | 0.009448 | 2.65E-09 | 0.489612 | 35.42841 |
|  | rs781663 | G | A | 0.058117 | 0.009753 | 2.54E-09 | 0.389744 | 35.50609 |
|  | rs11170820 | G | C | 0.089349 | 0.014913 | 2.08E-09 | 0.1519 | 35.89876 |
|  | rs79774308 | G | A | 0.126726 | 0.021071 | 1.81E-09 | 0.054899 | 36.17174 |
|  | rs77303550 | T | C | -0.07275 | 0.012067 | 1.65E-09 | 0.214902 | 36.34379 |
|  | rs12646335 | A | G | -0.05803 | 0.009559 | 1.27E-09 | 0.461238 | 36.85756 |
|  | rs9411465 | A | G | 0.066237 | 0.010863 | 1.08E-09 | 0.268052 | 37.17718 |
|  | rs651821 | T | C | -0.06199 | 0.010164 | 1.07E-09 | 0.651116 | 37.18992 |
|  | rs57301765 | A | G | 0.063197 | 0.01014 | 4.6E-10 | 0.346948 | 38.842 |
|  | rs11835398 | G | C | 0.062351 | 0.010002 | 4.55E-10 | 0.361396 | 38.86158 |
|  | rs13070927 | T | G | -0.07234 | 0.011543 | 3.68E-10 | 0.213807 | 39.27629 |
|  | rs145044106 | C | G | 0.150017 | 0.023545 | 1.87E-10 | 0.041673 | 40.59603 |
|  | rs11655024 | C | T | -0.08637 | 0.013532 | 1.74E-10 | 0.834191 | 40.7336 |
|  | rs1344541 | A | G | 0.099592 | 0.015492 | 1.29E-10 | 0.101993 | 41.32766 |
|  | rs73386640 | G | A | 0.092428 | 0.014296 | 1.01E-10 | 0.12433 | 41.80066 |
|  | rs4708876 | G | C | -0.09249 | 0.014287 | 9.55E-11 | 0.873705 | 41.91178 |
|  | rs9751370 | G | A | 0.064917 | 0.010002 | 8.56E-11 | 0.498724 | 42.12571 |
|  | rs9411378 | A | C | 0.069296 | 0.010585 | 5.89E-11 | 0.274618 | 42.8569 |
|  | rs61744384 | A | T | 0.064741 | 0.009818 | 4.28E-11 | 0.384325 | 43.48139 |
|  | rs12621411 | A | C | 0.070056 | 0.01041 | 1.7E-11 | 0.340682 | 45.28886 |
|  | rs2681492 | C | T | 0.067722 | 0.009767 | 4.09E-12 | 0.377659 | 48.07919 |
|  | rs442174 | A | T | 0.127648 | 0.018114 | 1.83E-12 | 0.072378 | 49.6586 |
|  | rs999675 | T | C | 0.067326 | 0.009509 | 1.44E-12 | 0.551686 | 50.13437 |
|  | rs34436970 | A | G | 0.099307 | 0.013845 | 7.36E-13 | 0.134378 | 51.44694 |
|  | rs1894401 | A | G | -0.10077 | 0.013811 | 2.97E-13 | 0.821821 | 53.22953 |
|  | rs67870673 | T | C | -0.07106 | 0.009627 | 1.57E-13 | 0.424487 | 54.48645 |
|  | rs903352 | C | A | -0.07459 | 0.009717 | 1.63E-14 | 0.618829 | 58.93318 |
|  | rs72874178 | A | G | -0.07718 | 0.010032 | 1.43E-14 | 0.362166 | 59.18877 |
|  | rs2738464 | C | G | 0.07872 | 0.010203 | 1.2E-14 | 0.685465 | 59.53334 |
|  | rs2296285 | A | T | 0.079307 | 0.009878 | 9.88E-16 | 0.384034 | 64.45416 |
|  | rs9521686 | C | T | 0.082908 | 0.010213 | 4.75E-16 | 0.690853 | 65.89673 |
|  | rs1443120 | G | C | 0.078821 | 0.009701 | 4.47E-16 | 0.413025 | 66.01618 |
|  | rs77335224 | T | C | -0.09334 | 0.011461 | 3.81E-16 | 0.238781 | 66.32994 |
|  | rs11107909 | T | C | -0.09402 | 0.011343 | 1.14E-16 | 0.232879 | 68.70681 |
|  | rs9295939 | G | A | 0.164702 | 0.018364 | 3E-19 | 0.070084 | 80.44011 |
|  | rs10895529 | G | C | -0.09069 | 0.00991 | 5.64E-20 | 0.367605 | 83.74022 |
|  | rs57224109 | G | A | 0.169562 | 0.018526 | 5.56E-20 | 0.069718 | 83.7701 |
|  | rs9395214 | G | C | 0.112544 | 0.012234 | 3.6E-20 | 0.802938 | 84.62953 |
|  | rs34166180 | A | C | -0.09088 | 0.009777 | 1.47E-20 | 0.551859 | 86.40375 |
|  | rs6841473 | T | C | 0.094958 | 0.010199 | 1.27E-20 | 0.313352 | 86.68428 |
|  | rs75346744 | G | A | 0.18474 | 0.01983 | 1.21E-20 | 0.061467 | 86.7896 |
|  | rs28709375 | G | A | 0.095031 | 0.009483 | 1.22E-23 | 0.542939 | 100.4333 |
|  | rs2327429 | C | T | -0.0992 | 0.00946 | 9.98E-26 | 0.517734 | 109.9628 |
|  | rs3858704 | G | A | -0.16331 | 0.010142 | 2.45E-58 | 0.677751 | 259.2901 |
|  | rs10757274 | G | A | 0.169942 | 0.009448 | 2.43E-72 | 0.475325 | 323.56 |

SNP: single nucleotide polymorphism; EA: effect allele; OA: other allele; Beta was obtained by allele-related effects; SE: standard error; Eaf: effect allele frequency. Beta, SE, and Pval are SNP summary statistics; LDL-C: Low-density-lipoprotein cholesterol; CAD: Coronary artery disease; HCC: Hepatocellular carcinoma.


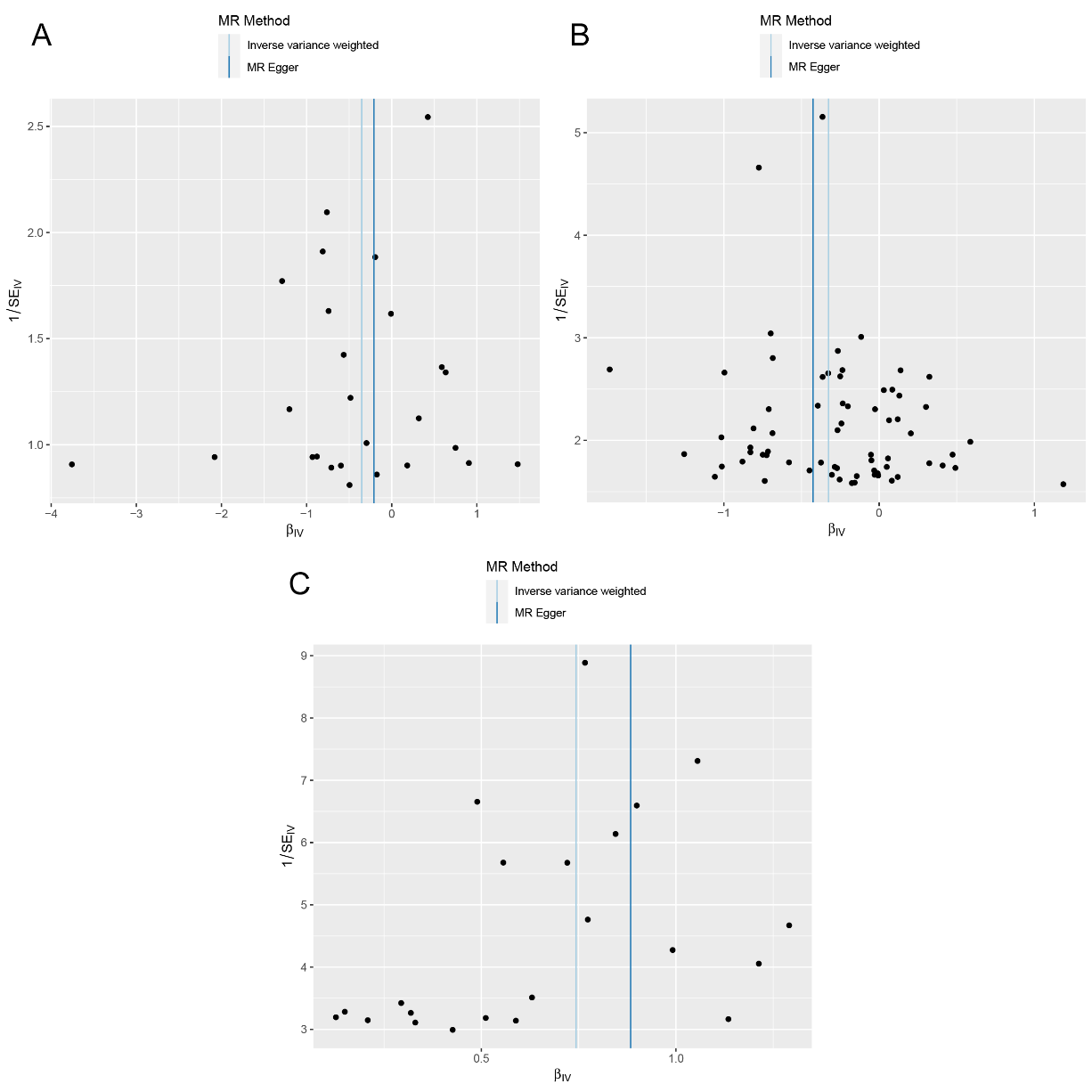


**Figure S1** Funnel plots for heterogeneity test. (A) LDL-C on HCC. (B) CAD on HCC. (C) LDL-C on CAD.
